# NLIP and HAD-like Domains of Pah1 and Lipin 1 Phosphatidate Phosphatases Are Essential for Their Catalytic Activities

**DOI:** 10.3390/molecules26185470

**Published:** 2021-09-08

**Authors:** Wei-Hsin Hsu, Yi-Hao Huang, Pin-Ru Chen, Lu-Sheng Hsieh

**Affiliations:** Department of Food Science, Tunghai University, No. 1727, Section 4, Taiwan Boulevard, Xitun District, Taichung 40704, Taiwan; g07621010@go.thu.edu.tw (W.-H.H.); g09621001@thu.edu.tw (Y.-H.H.); s07620233@thu.edu.tw (P.-R.C.)

**Keywords:** *Saccharomyces cerevisiae*, Pah1 phosphatidate phosphatase, N-terminal Lipin (NLIP) domain, C-terminal Lipin (CLIP)/haloacid dehalogenase (HAD)-like domain, Lipin 1 phosphatidate phosphatase, thioredoxin

## Abstract

*Saccharomyces cerevisiae* Pah1 phosphatidate phosphatase (PAP) catalyzes the dephosphorylation of phosphatidate to yield diacylglycerol, controlling phospholipids and triacylglycerol metabolisms. Pah1 and human Lipin 1 are intrinsically disordered proteins with 56% and 43% unfolded regions, respectively. Truncation analysis of the conserved and non-conserved regions showed that N- and C-conserved regions are essential for the catalytic activity of Pah1. PAP activities can be detected in the conserved N-terminal Lipin (NLIP) domain and C-terminal Lipin (CLIP)/haloacid dehalogenase (HAD)-like domain of Pah1 and Lipin 1, suggesting that the evolutionarily conserved domains are essential for the catalytic activity. The removal of disordered hydrophilic regions drastically reduced the protein solubility of Pah1. Thioredoxin is an efficient fusion protein for production of soluble NLIP–HAD recombinant proteins in *Escherichia coli.*

## 1. Introduction

**Figure 1:**
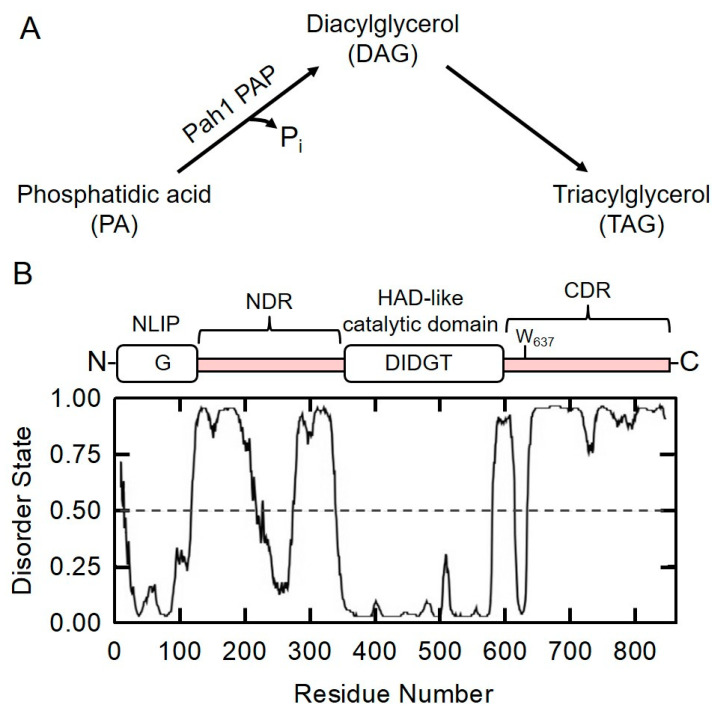
The reaction catalyzed by the yeast Pah1 phosphatidate phosphatase is shown (**A**). Pah1 PAP catalyzes the dephosphorylation of phosphatidic acid (PA) to produce diacylglycerol (DAG) for the biosynthesis of triacylglycerol (TAG). The domain structure and prediction of intrinsically disordered regions in Pah1 PAP are presented (**B**). N-terminal Lipin (NLIP) and haloacid dehalogenase (HAD)-like domains are in the N-terminus and middle of Pah1, respectively; DIDGT is the catalytic motif in the HAD-like domain. Besides the conserved regions, there are two non-conserved regions, namely N-terminal disordered region (NDR, residues 99–359) and C-terminal disordered region (CDR, residues 592–862). The Pah1 sequence was analyzed using the DISOPRED3 algorithm (http://bioinf.cs.ucl.ac.uk/psipred/) (accessed on 1 March 2020).

The phosphatidate phosphatase (PAP, EC 3.1.3.4) reaction was initially reported in Kennedy’s lab [1], catalyzing the penultimate reaction of triacylglycerol (TAG) synthesis by converting phosphatidate (PA) to diacylglycerol (DAG) (Figure 1A). The DAG synthesized by a PAP reaction can also be used for the synthesis of phosphatidylcholine (PC) and phosphatidylethanolamine (PE) via the Kennedy pathway [2,3]. In the yeast *Saccharomyces cerevisiae*, Carman and coworkers discover four proteins exhibiting PA phosphatase activities, namely App1 [4], Dpp1 [5], Lpp1 [6], and Pah1 [7]; however, only Pah1 PAP is involved in storage lipid triacylglycerol synthesis [7,8]. N-terminal Lipin (NLIP) and HAD-like, also known as C-terminal Lipin (CLIP), catalytic domains are conserved in Pah1 (Figure 1B), and the DIDGT catalytic motif is presented in the HAD-like catalytic domain [7,8]. The conserved Trp-637 within the WRDPLVDID domain of Pah1 is non-essential for the PAP activity but is required for its functions in vivo [9]. Pah1 PAP activity plays important roles in the formation of lipid droplets [10,11,12,13], and maintains vacuole homeostasis and membrane fusion [14]. Altering lipid metabolism by eliminating Pah1 PAP activity shows some potential biotechnological applications such as increased β-carotene production in *Saccharomyces cerevisiae* [15], enhanced triterpenoid production [16], and membrane protein expression [17] in *Yarrowia lipolytica.*

Pah1 is one of the highly regulated enzymes in lipid metabolism [2,18]. Phosphorylation/dephosphorylation regulates the subcellular localization, protein abundance, and PAP activity of Pah1 [19,20]. The newly synthesized Pah1 protein is localized in the cytosol, and is phosphorylated by multiple protein kinases [21,22,23] such as CDC28-cyclin B [24], Pho85-Pho80 [19], protein kinase A [25], protein kinase C [26], casein kinase II [27], and casein kinase I [28]. Phosphorylated Pah1 is recruited to the endoplasmic reticulum (ER) membrane to be dephosphorylated by the Nem1-Spo7 protein phosphatase [21,29,30]. Dephosphorylated Pah1 is activated and mediated the PAP reaction on the ER membrane [7]. Phosphorylation status also controls the turnover of Pah1 via ubiquitin-independent proteasome-dependent degradation [20,31]. Dephosphorylated Pah1 is readily degraded by the 20S proteasome, but not the 26S proteasome [20]. Unfolded regions of Pah1 protein initiate the degradation of the 20S proteasome, whereas the compacted HAD-like domain is inaccessible for the 20S proteasome [20].

Human Pah1 orthologs, Lipins, are encoded by a multi-gene family, namely *LPIN1*, *LPIN2*, and *LPIN3* [32,33]. *Escherichia coli*-expressed un-ubiquitinated recombinant human Lipin 1 protein is also subjected to degradation by the yeast 20S proteasome but not by the 26S proteasome [20]. Pah1 and Lipins are predicted to be intrinsically disordered proteins, sharing an evolutionarily conserved proteasomal degradation mechanism [20]. Besides the ubiquitin-independent pathway, Lipin 1 and 2 have been shown to be regulated by ubiquitination [34,35,36,37]. Degradation of Lipin 1 via the ubiquitin–proteasome system mediates lipogenesis, fibrogenesis, and TGF-β signaling in liver cells [34,35]. Several studies have shown that Lipin 1 deficiency is linked to recurrent rhabdomyolysis in human children [38,39,40,41,42], suggesting that Lipin 1 PAP activity is involved in the acute syndromes of rhabdomyolysis.

*Tetrahymena thermophila* Pah/Lipin homologs are encoded by *TtPAH1* and *TtPAH2* genes [12,43]. In terms of protein primary structure, TtPah1 protein is about 96 kDa which is comparable to yeast Pah1 and human Lipin 1, whereas TtPah2 is a low molecular weight protein of 37 kDa with fused NLIP and HAD-like conserved domains [43]. PAP activity of TtPah2 is detectable; however, TtPah2 cannot complement lipid droplet biogenesis and ER morphology defects caused by the lack of Pah1 PAP in yeast cells [43]. A great breakthrough of the first TtPah2 crystal structure was recently reported by Khayyo et al. (2020) [44]. In addition, mouse Lipin 2 NLIP–CLIP fusion (deletion residues between 94 and 627) is an active PAP enzyme [44]. Thus far, there is no 3D structure of full-length Pah1/Lipin proteins because they are not readily expressed heterologously due to their molecular mass (>95 kDa). To find out the minimum required regions for Pah1 PAP activity, we conducted deletion analysis combined with PAP activity determinations for better understanding of the functions of conserved NLIP and HAD-like domains as well as non-conserved/unfolded regions in the yeast Pah1.

## 2. Results and Discussion

### 2.1. Prediction of Intrinsically Disordered Regions in Pah1 by DISPRED3 Algorithm

The domain structure of Pah1 is shown in Figure 1B. N-terminal Lipin (NLIP, residues 1–98) and haloacid dehalogenase (HAD, residues 360–591)-like domains are evolutionarily conserved regions. The Pah1 sequence is analyzed using the DISOPRED3 algorithm [45], and the ratio of disorder states indicates ordered/folded and disordered/unfolded regions, respectively. Pah1 is predicted as a highly unfolded protein with 56% disordered regions. The major non-conserved region in between NLIP and HAD-like domains is designated as the N-terminal disordered region (NDR, residues 99–359), and the non-conserved region adjacent to HAD-like domains is designated as the C-terminal disordered region (CDR, residues 592–862). In the CDR, Trp-637 within the WRDPLVDID domain of Pah1 is predicted to be ordered/folded. Although the small TtPah2 crystal structure was reported last year [44], full-sized Pah/Lipin proteins were not successfully crystalized, presumably due to a high degree of disordered regions that lack a rigid three-dimensional structure [46]. Phosphorylation sites identified in Pah1 are mainly localized in disordered regions, e.g., casein kinase II (CKII) phosphorylates Pah1 on Thr-170, Ser-250, Ser-313, Ser-705, Ser-814, and Ser-818 [27]. All six CKII phosphorylation sites are located in the NDR and CDR of Pah1 [27]. The structure of the unfolded region is generally recognized as more flexible than that of the folded region, creating more opportunities for protein–protein interactions [47]. 

### 2.2. HAD-like Domain Is Essential for Pah1 Catalytic Activity

**Figure 2:**
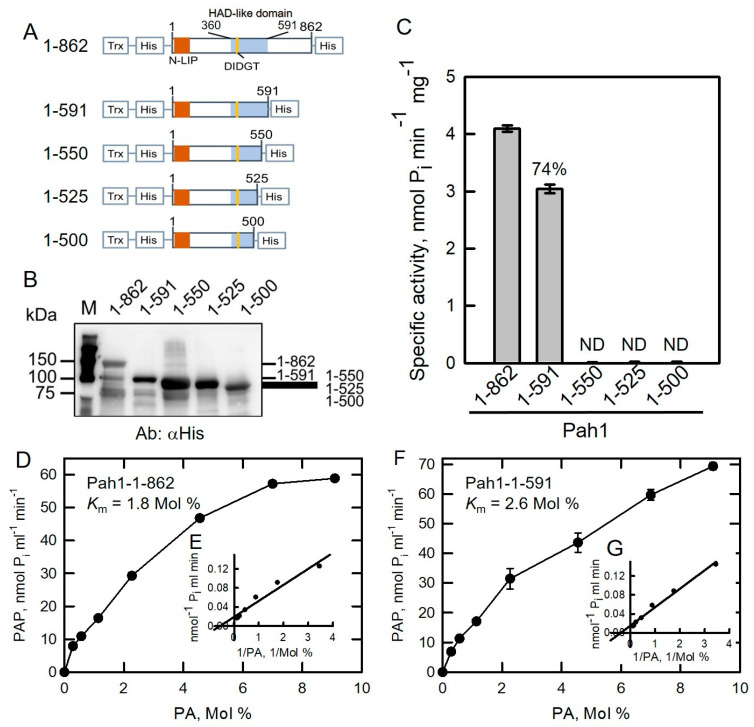
HAD-like domain is essential for Pah1 catalytic activity. (**A**) The full-length and truncated Pah1 proteins fused with thioredoxin (Trx) and His_6_-tags at both termini. (**B**) Pah1 truncations were expressed in *E. coli* and purified by Ni-NTA resin. Proteins were subjected to SDS-PAGE followed by Western blotting analysis with the anti-His antibodies. (**C**) PAP activities were measured in Pah1 truncations. (**D**) Substrate saturation curve and Lineweaver–Burk double reciprocal plot (**E**) of the initial rate result of Pah1-1-862. (**F**) Substrate saturation curve and Lineweaver–Burk double reciprocal plot (**G**) of the initial rate result of Pah1-1-591. The data are the average of triplicate experiments ± S.D. (error bars). ND, not detected.

In general, ordered and disordered regions of Pah1 are hydrophobic and hydrophilic, respectively. Although some truncated Pah1 proteins were expressed in *E. coli* and used for an in vitro proteasomal degradation assay [20], PAP activity analysis has not been performed before. Removal of disordered regions of Pah1, NLIP and HAD-like domain fusion (1-98-360-591), was expressed in E. coli but could only be detected by anti-His antibodies in the insoluble inclusion body fraction [20], indicating that hydrophilic disordered regions are required for maintaining Pah1 solubility. Herein, we used another strategy to merge a thioredoxin (Trx) protein at the N-terminus of the fusion protein to increase its protein solubility in Pah1 truncations [48,49]. We also tried other fusion proteins such as maltose-binding protein and glutathione S-transferase; however, thioredoxin fusion protein gave us the best result. In addition, six histidine residues are tagged at both termini to facilitate affinity purification of recombinant proteins (Figure 2A). The full-length *PAH1* sequence was inserted into a pET32b plasmid (Table 1) and its accuracy was confirmed with restriction enzyme digestions (Figure S1A). Accordingly, four truncated Pah1 fragments were subcloned into a pET32b plasmid and then confirmed with restriction enzyme digestions (Figure S1B–E). All plasmids were further verified by DNA sequencing followed by expression in *E. coli.*

Yeast expressed Pah1-1-591 is an active PAP enzyme [9]. Based on the result, we first examined the importance of the HAD-like domain by eliminating its amino acid residues to generate Pah1-1-591, 1-550, 1-525, and 1-500 truncations (Figure 2). The active full-length Pah1 protein was used as control. Purified full-length and truncated Pah1 proteins were separated using SDS-PAGE followed by Western blotting analysis with anti-His antibodies (Figure 2B). All full-length and truncated Pah1 were purified and detected by antibodies. PAP activity was examined in full-length and truncated Pah1 proteins (Figure 2C). Pah1-1-591 truncation retained 74% PAP activity compared with full-length Pah1-1-862. This result further confirmed that the conserved Trp-637 is non-essential for PAP activity as reported previously [9]. In yeast, the lack of Pah1 function in the *pah1*Δ mutant is lethal at 37 °C [8,9,20]. Several mutations of Trp-637, such as W637A, W637E, and W637R, and 1-591 truncated Pah1 protein exhibited PAP activities but could not complement the temperature-sensitive phenotype in the *pah1*Δ mutant, suggesting that the catalytic activity of Pah1 is not sufficient for completing its functions in vivo [9]. Except Pah1-1-591, the other three truncations, 1-550, 1-525, and 1-500, that delete partial HAD-like domain amino acid residues exhibited null-PAP activity, indicating that the HAD-like domain of Pah1 is essential for its PAP activity.

Kinetic parameters were measured and compared in wild type Pah1-1-862 and truncated Pah1-1-591 proteins. The *K*_m_ value for Pah1-1-862 toward PA was calculated as 1.8 Mol % (Figure 2D,E) which was lower than that of Pah1-1-591 (2.6 Mol %, Figure 2F,G), indicating that full-length Pah1-1-862 showed better affinity toward its substrate PA. The *k*_cat_ value of Pah1-1-862 toward PA was calculated as 7.22 s^−1^ (Table 2), which was about 2.2-fold higher than that of Pah1-1-591 (3.25 s^−1^). In terms of *k*_cat_/*K*_m_ value, the overall catalytic property of Pah1-1-862 was 3.2-fold higher than that of Pah1-1-591 (Table 2). Taken together, removal of the C-terminal non-conserved region decreases affinity to the substrate as well as overall catalytic function.

### 2.3. Disordered Regions Are Responsible for the Solubility of Pah1

**Figure 3:**
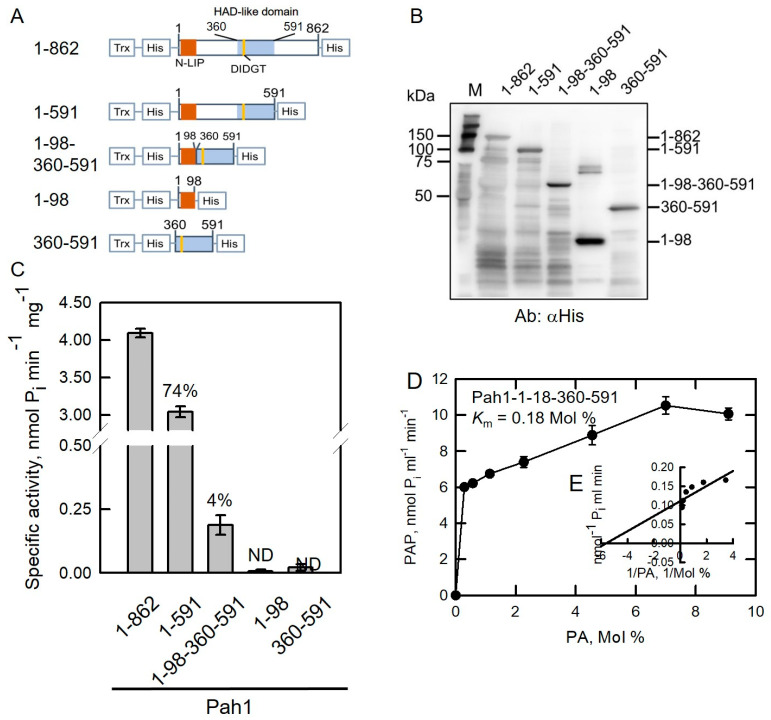
NLIP domain is essential for Pah1 catalytic activity. (**A**) The full-length and truncated Pah1 proteins fused with thioredoxin (Trx) and His_6_-tags at both termini. (**B**) Pah1 truncations were expressed in *E. coli* and purified by Ni-NTA resin. Proteins were subjected to SDS-PAGE followed by Western blotting analysis with the anti-His antibodies. (**C**) PAP activities were measured in Pah1 truncations. (**D**) Substrate saturation curve and Lineweaver–Burk double reciprocal plot (**E**) of the initial rate result of Pah1-1-98-360-591. The data are the average of triplicate experiments ± S.D. (error bars). ND, not detected.

As shown previously, Pah1-1-591 retained 74% PAP activity. We further removed the NDR (residues between 99 and 359) to generate the Pah1-1-98-360-591 truncation (Supplementary Material Figure S2A and Figure 3A). Directly fused NLIP and HAD truncation protein is completely insoluble [20]. To facilitate protein purification and increase protein solubility, His-tag and thioredoxin were fused as indicated in Figure 3A. In addition, Pah1-1-98 (Figure S2B and Figure 3A) and Pah1-360-591 truncations were generated (Figure S2C and Figure 3A). 

As shown in Figure 3B, purified full-length and truncated Pah1 proteins migrated to “relative” positionsand were detected by anti-His antibodies. PAP activities were compared in these proteins. Pah1-1-98-360-591 retained a small but unignorable amount of PAP activity of 4% compared with full-length Pah1 (Figure 3C). The other two truncations, Pah1-1-98 and Pah1-360-591, exhibited null PAP activity (Figure 3C). Pah1-1-98-360-591 fused with thioredoxin was expressed and purified as a soluble active PAP protein, suggesting that conserved NLIP and HAD-like domains are essential for Pah1 catalytic activity. Kinetic parameters were measured in truncated Pah1-1-98-360-591 proteins. The *K*_m_ value for Pah1-1-98-360-591 toward PA was estimated as 0.18 Mol % (Figure 3D,E) which was much lower than Pah1-1-862 (1.8 Mol %) and Pah1-1-591 (2.6 Mol %), presumably because Pah1-1-98-360-591 is a less active PAP enzyme. The *k*_cat_ value of Pah1-1-98-360-591 toward PA was calculated as 0.74 s^−1^ (Table 2), which was about 10-fold lower than that of Pah1-1-862 (7.22 s^−1^). In terms of *k*_cat_/*K*_m_ value (Table 2), the overall catalytic property of Pah1-1-98-360-591 (4.11 s^−1^ Mol %^−1^) was comparable to that of Pah1-1-862 (4.01 s^−1^ Mol %^−1^), suggesting that the truncation Pah1-1-98-360-591 with only 330 amino acids (38% of full-length protein, 862 amino acids) is still a functional active PAP enzyme. 

### 2.4. Prediction of Intrinsically Disordered Regions in Human Lipin 1-α by DISPRED3 Algorithm

**Figure 4:**
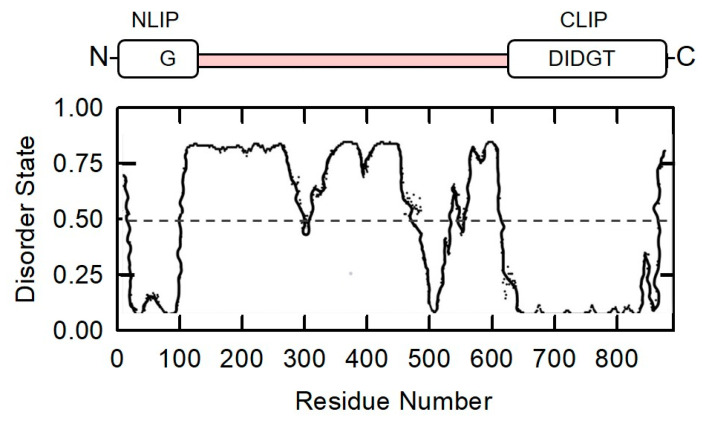
The domain structure and prediction of intrinsically disordered regions in Lipin 1-α are presented. NLIP and CLIP domains are in the N- and C-termini of Lipin 1-α, respectively. DIDGT is the catalytic motif in CLIP domain. Protein sequence was analyzed using the DISOPRED3 algorithm (http://bioinf.cs.ucl.ac.uk/psipred/) (accessed on 15 May 2021).

The domain structure of Lipin 1-α is shown in Figure 4. NLIP (residues 1–114) and CLIP (residues 601–890) domains are evolutionarily conserved regions. The Lipin 1-α sequence is analyzed using the DISOPRED3 algorithm which the disorder states indicate ratios of ordered/folded and disordered/unfolded regions. Lipin 1-α is predicted as a highly unfolded protein with 43% disordered regions. 

### 2.5. NLIP–HAD-like Truncation of Human Lipin 1 Is Active

**Figure 5:**
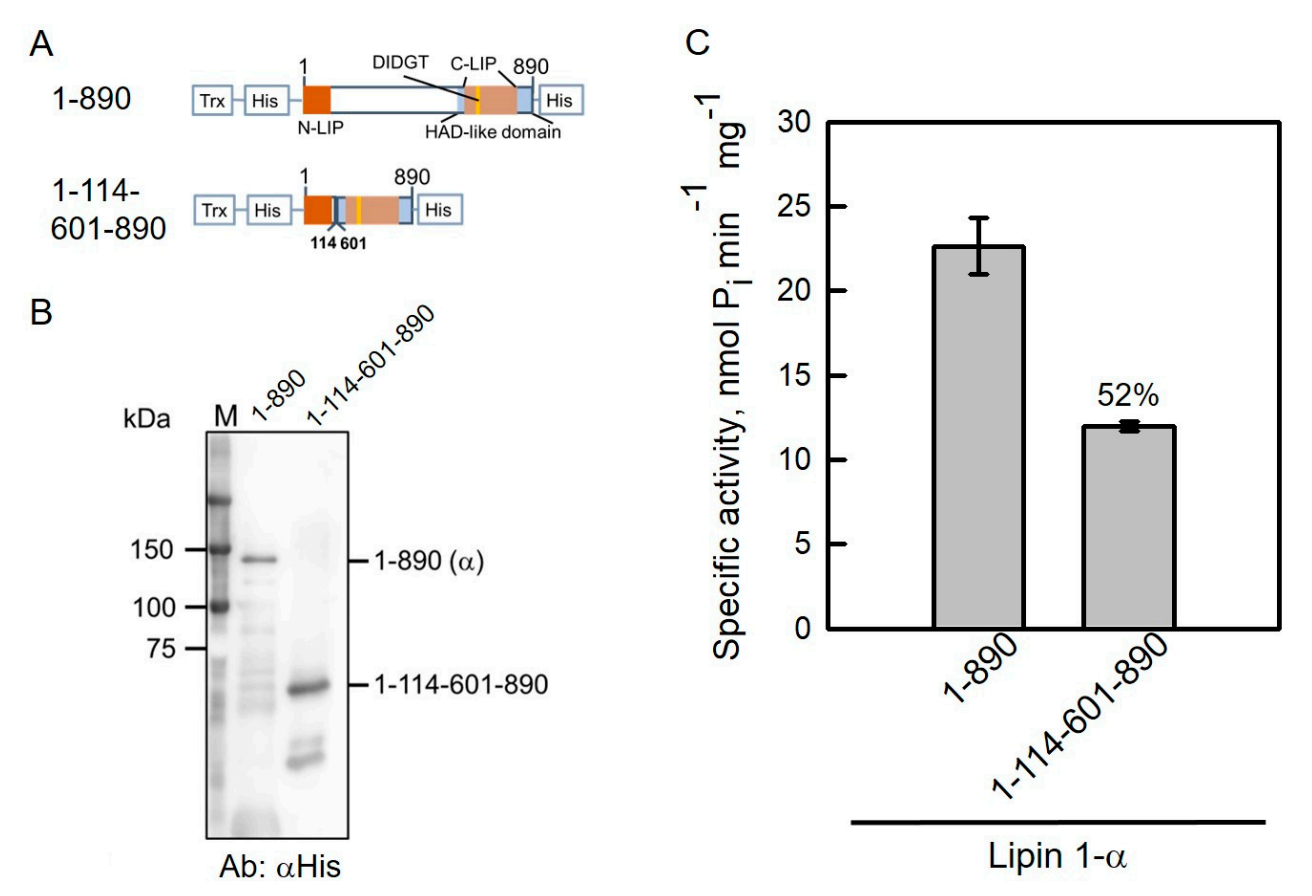
NLIP and HAD-like domains are essential for Lipin 1 catalytic activity. (**A**) The full-length and truncated Lipin 1-α proteins fused with thioredoxin (Trx) and His_6_-tags at both termini. (**B**), Lipin 1-α truncations were expressed in *E. coli* and purified by Ni-NTA resin. Proteins were subjected to SDS-PAGE followed by Western blotting analysis with the anti-His antibodies. (**C**) PAP activities were measured in Pah1 truncations. The data are the average of triplicate experiments ± S.D. (error bars).

Lipin 1-α has been shown to be more active than Lipin 1-β and Lipin 1-γ in vitro [33]. The conserved regions, NLIP and CLIP domains, are localized at both termini of Lipin 1-α (Figure 5A). To facilitate Lipin 1 protein purification and increase protein solubility, His-tag and thioredoxin were fused as indicated in Figure 5A. The full-length *LPIN1-α* sequence was inserted into pET32b plasmid and its accuracy was confirmed with restriction enzyme digestions (Table 1; Figure S3A). Accordingly, NLIP and CLIP fusion of Lipin 1 (pET32b-Lipin1-α-1-114-601-890) was generated by PCR-mediated deletion of the pET32b-Lipin 1 plasmid followed by restriction enzyme digestions (Figure S3B). Both plasmids were further verified by DNA sequencing and expressed in *Escherichia coli*.

As shown in Figure 5B, purified full-length and truncated Lipin 1-1-114-601-890 proteins migrated to relative positions and were detected by anti-His antibodies. PAP activities were compared in full-length and truncated Lipin 1 proteins. Lipin 1-1-114-601-890 retained 52% PAP activity compared with full-length Lipin 1-α (Figure 5C). Additionally, Lipin 1-1-114-601-890 fused with thioredoxin was expressed and purified as a soluble active protein, suggesting that conserved NLIP and CLIP domains are essential for Lipin 1 catalytic activity.

## 3. Materials and Methods

### 3.1. Reagents

Bio-Rad protein assay dye reagent [50], iProof DNA polymerase, Nuvia^TM^ IMAC resin, Precision plus protein dual color standards, and reagents for protein electrophoresis were mainly purchased from Bio-Rad, Hercules, CA, USA. Restriction endonucleases, PrimeSTAR DNA polymerase, and the In-Fusion HD cloning kit were obtained from Takara, Japan. SeaKem^®^ LE Agarose was purchased from Lonza, Morristown, NJ, USA. The Presto^TM^ Mini Plasmid kit and GenepHlow^TM^ Gel/PCR kit were supplied by Geneaid, New Taipei City, Taiwan. Goat anti-mouse IgG H&L (HRP, ab6789) and a colorimetric phosphate assay kit—PiColorLock^TM^ (ab27004) were obtained from Abcam, Cambridge, UK. Immobilon PVDF membranes, Western Chemiluminescent HRP substrate (ECL), and anti-6x His tag monoclonal antibodies (Cat. # 05-949, Lot # 2869961) were purchased from Merck Millipore, Burlington, MA, USA. Di-C18:0 PA (Lot # 830865P-25MG-C-045) was obtained from Avanti Polar Lipids, Alabaster, AL, USA. Oligonucleotide synthesis and DNA sequencing were carried out by Tri-I Biotech, New Taipei City, Taiwan. All other chemicals were reagent grade.

### 3.2. Molecular Manipulations and Construction of Plasmids

The full-length yeast *PAH1* and human *LPIN-1-α* sequences were amplified by polymerase chain reaction (PCR) using pGH313 [7] and pGH322 [33] plasmids as templates, respectively. PCR conditions were conducted under the standard 30-cycle reaction, 98 °C for 30 s, 60 °C for 15 s, and 72 °C for 60 s, followed by a final extension for 5 min at 72 °C using PrimeSTAR proofreading DNA polymerase (Takara, Shiga, Japan). DNA fragments of *PAH1* and *LPIN-1-α* were individually subcloned into the pET32b plasmid using the In-Fusion HD Cloning kit (Takara, Japan) to construct pET32b-ScPah1-1-862 and pET32b-Lipin 1-α-1-890 plasmids (Table 1). Plasmids were verified by restriction endonuclease digestion, *Kpn*I and/or *Xho*I, followed by DNA sequencing. The deletion of *PAH1* and *LPIN-1-α* codons was performed by In-Fusion cloning combined with the PCR method with specific primers to construct the truncation plasmids listed in Table 1. All plasmids were confirmed with restriction enzyme digestion and DNA sequencing.

### 3.3. Escherichia coli Strains and Protein Expression Conditions

*E. coli* DH5α was used for the proliferation of plasmids, and BL21(DE3) was transformed for the expression of the yeast Pah1 and human Lipin 1-α truncations as described previously [20,49,51]. *E. coli* DE3 strains carrying the plasmids (Table 1) were grown at 37 °C in 250 mL of Luria–Bertani (LB, 1% tryptone, 0.5% yeast extract, 1% NaCl) medium containing 100 μg/mL ampicillin. To induce protein expression, a final concentration of 1.0 mM isopropyl-β-d-thiogalactoside (IPTG) was added and then incubated at 30 °C for 3 h with vigorous shaking. Cells were harvested by centrifugation at 6000× *g* for 10 min, and stored at −20 °C until use. 

### 3.4. Purification of Recombinant Proteins by Affinity Chromatography

*E. coli* cells were resuspended in lysis buffer (50 mM Tris-HCl, 150 mM NaCl, 10 mM imidazole, 1 mM phenylmethanesulfonyl fluoride (PMSF), pH 7.4) and disrupted by sonication (Ultrasonic Processors, Sonicator 3000, Misonix, Farmingdale, NY, USA) [49,51]. Cell lysates were added into an open column containing an appropriate amount of Ni-charged resin (Bio-Rad, USA). Proteins were fractionated by eluting with buffers containing 10, 50, 125, 250, and 500 mM imidazole. All the purification steps were performed in a 4 °C cold room.

### 3.5. SDS-Polyacrylamide Gel Electrophoresis and Western Blotting Analysis

Purified full-length or truncated proteins were subjected to polyacrylamide gel electrophoresis, generally on 10% SDS gels using a Mini-PROTEAN Tetra Cell system (Bio-Rad, USA). Proteins were then transferred onto Immobilon PVDF membranes (Merck Millipore, USA) using a Mini Trans-Blot Electrophoretic Transfer Cell system (Bio-Rad, USA). Membranes were probed with mouse anti-His antibodies (1 μg/mL) and then horseradish peroxidase-conjugated goat anti-mouse IgG antibodies (1:5000 dilution) following a standard protocol [20,27]. Immune complexes were monitored by the enhanced chemiluminescent (ECL) substrate; gel images were captured by a ChemiLITE Chemiluminescent Imaging System (Cleaver Scientific, Rugby, UK). Protein signals on blot images were analyzed and quantified using ImageJ software (NIH, Rockville, MD, USA).

### 3.6. PAP Activity Assay

Protein quantitation was determined by a dye-binding assay [50] using bovine serum albumin as standard. The PAP reaction mixture in a total volume of 100 μL contained 50 mM Tris-HCl (pH 7.5), 1 mM MgCl_2_, 0.2 mM PA, 2 mM Triton X-100, and an aliquot of enzyme protein. Pah1 [7] and Lipin 1 [33] activities were measured for 20 min at 30 °C and 37 °C. The PAP reaction mixture was combined with 25 μL P_i_ColorLock reagent for 5 min, and then 2 μL stabilizer was added for 30 min, measuring the absorbance at 650 nm. PAP-specific activity was defined as the amount of enzyme that catalyzed the formation of 1 nmol of P_i_ per minute per mg of protein.

### 3.7. PAP Enzyme Kinetic

To determine the kinetic parameters of Pah1 truncations, the concentrations of PA were varied from 0 to 9.09 Mol %. A substrate saturation curve was obtained after a 10 min incubation [7,51]. A double reciprocal plot was adapted for calculation of the *K*_m_ and *k*_cat_ values [52]. Enzyme kinetic curves were made and statistical analysis was performed using SigmaPlot software (SigmaPlot 11th version, Chicago, IL, USA). 

## 4. Conclusions

Pah1 and Lipin 1 proteins are predicted to be intrinsically disordered proteins by the DISPRED3.0 algorithm. Systematically removing the unstructured regions combined with a PAP activity assay shows that disordered regions are non-essential for the catalytic activities but are required to maintain Pah1/Lipin 1 protein solubility. Evolutionarily conserved NLIP and CLIP domains are essential for the catalytic activity of Pah1 and Lipin 1 proteins. Overexpressed thioredoxin-fused Pah1 truncated protein is a candidate for crystal structure determination.

## Figures and Tables

**Table 1:** List of plasmids used in this study.

Plasmid	Relevant Characteristics	Source/Ref.
pGH313	Full-length *PAH1* (1-862) coding sequence inserted into pET15b	[7]
pET32b	*E. coli* expression vector with thioredoxin (Trx) fusion protein and both N-terminal and C-terminal His_6_-tag fusions	Novagen
pET32b-ScPah1-1-862	Full-length *PAH1* (1-862) coding sequence inserted into pET32b	This study
pET32b-ScPah1-1-591	*PAH1* (1-591 truncation) inserted into pET32b	This study
pET32b-ScPah1-1-550	*PAH1* (1-550 truncation) inserted into pET32b	This study
pET32b-ScPah1-1-525	*PAH1* (1-525 truncation) inserted into pET32b	This study
pET32b-ScPah1-1-500	*PAH1* (1-500 truncation) inserted into pET32b	This study
pET32b-ScPah1-1-98	*PAH1* (1-98 truncation) inserted into pET32b	This study
pET32b-ScPah1-1-98-360-591	*PAH1* (1-98-360-591 truncation) inserted into pET32b	This study
pET32b-ScPah1-360-591	*PAH1* (360-591 truncation) inserted into pET32b	This study
pGH322	Full-length human *LPIN1*-α (1-890) coding sequence inserted into pET28b	[33]
pET32b-Lipin1-α-1-890	Full-length human *LPIN1*-α (1-890) coding sequence inserted into pET32b	This study
pET32b-Lipin1-α-1-114-601-890	*LPIN1*-α (1-114-601-890 truncation) inserted into pET32b	This study

**Table 2:** Kinetic parameters of full-length and truncations of Pah1.

Protein	*K*_m_ (Mol %)	*k*_cat_ (s^−1^)	*k*_cat_/*K*_m_ (s^−1^ Mol %^−1^)
Pah1-1-862	1.8	7.22	4.01
Pah1-1-591	2.6	3.25	1.25
Pah1-1-98-360-591	0.18	0.74	4.11

## Data Availability

Data are contained in the main article and Supplementary Materials.

## Supplementary Materials

The following are available online at. Figure S1: Confirmed plasmid accuracy by restriction enzyme digestion. Figure S2: Confirmed plasmid accuracy by restriction enzyme digestion. Figure S3: Confirmed plasmid accuracy by restriction enzyme digestion.

## Sample Availability

Samples of the compounds are not available from the authors.
