# Supplementary material for: NLIP and HAD-like Domains of Pah1 and Lipin 1 Phosphatidate Phosphatases Are Essential for Their Catalytic Activities
Source: Molecules. 2021 Sep 8;26(18):5470. doi: 10.3390/molecules26185470 (PMC8470223; doi:10.3390/molecules26185470)
Supplement: Supplementary file 1 [file molecules-26-05470-s001.zip › molecules-1353020-supplementary.pdf]

NLIP and HAD-like Domains of Pah1 and Lipin 1 Phosphatidate Phosphatases Are Essential for Their Catalytic Activities

Wei-Hsin Hsu, Yi-Hao Huang, Pin-Ru Chen and Lu-Sheng Hsieh*

Department of Food Science, Tunghai University, No. 1727, Section 4, Taiwan Boulevard, Xitun District, Taichung 40704, Taiwan, ROC. E-mail: g07621010@go.thu.edu.tw (W.H.H.), g09621001@thu.edu.tw (Y.H.H.), s07620233@thu.edu.tw (P.R.C)

*Corresponding author E-mail: lshsieh@thu.edu.tw (L.S.H.)

Tel.: 886-4-23590121#37331

Supplementary Materials

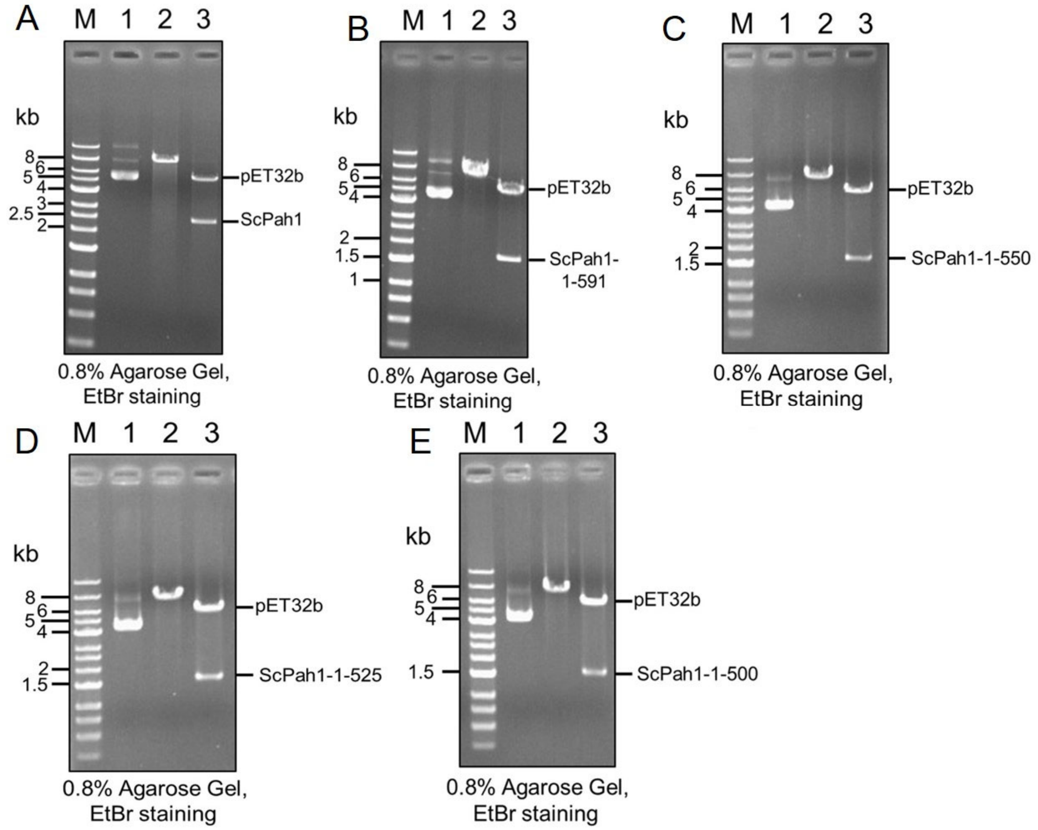

Figure S1. Confirm plasmid accuracy by restriction enzyme digestion. *A*, lane 1, pET32b-ScPah1 (1-862) plasmid only; lane 2, plasmid digested with *Kpn*I; lane 3, plasmid digested with *Kpn*I and *Xho*I. *B*, lane 1, pET32b-ScPah1-1-591 plasmid only; lane 2, plasmid digested with *Kpn*I; lane 3, plasmid digested with *Kpn*I and *Xho*I. *C*, lane 1, pET32b-ScPah1-1-550 plasmid only; lane 2, plasmid digested by *Kpn*I; lane 3, plasmid digested with *Kpn*I and *Xho*I. *D*, pET32b-ScPah1-1-525 plasmid only; lane 2, plasmid digested with *Kpn*I; lane 3, plasmid digested with *Kpn*I and *Xho*I. *E*, pET32b-ScPah1-1-500 plasmid only; lane 2, plasmid digested with *Kpn*I; lane 3, plasmid digested with *Kpn*I and *Xho*I. DNA fragments were separated by 0.8% agarose gel electrophoresis followed by EtBr staining. Images were captured by a Gel Doc XR+ Imaging Systems (Bio-Rad, USA).

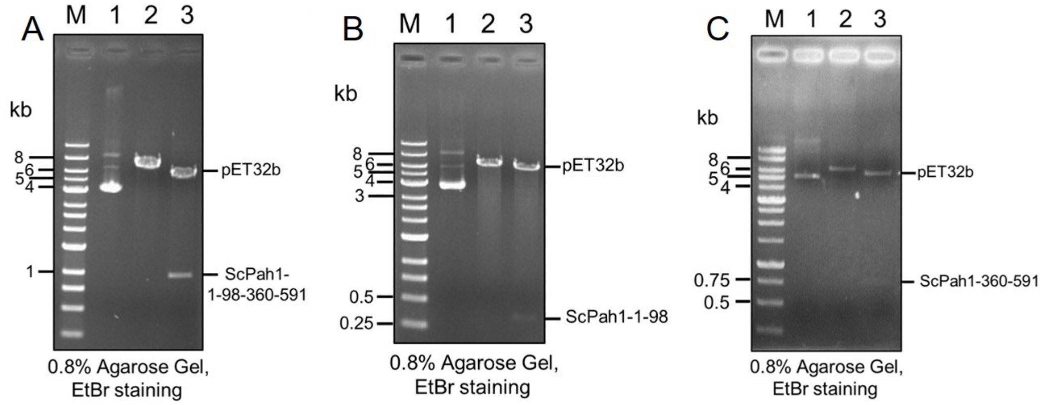

Figure S2. Confirm plasmid accuracy by restriction enzyme digestion. *A*, lane 1, pET32b-ScPah1 (1-98-360-591) plasmid only; lane 2, plasmid digested with *Kpn*I; lane 3, plasmid digested with *Kpn*I and *Xho*I. *B*, lane 1, pET32b-ScPah1-1-98 plasmid only; lane 2, plasmid digested with *Kpn*I; lane 3, plasmid digested with *Kpn*I and *Xho*I. *C*, lane 1, pET32b-ScPah1-360-591 plasmid only; lane 2, plasmid digested with *Kpn*I; lane 3, plasmid digested with *Kpn*I and *Xho*I. DNA fragments were separated by 0.8% agarose gel electrophoresis followed by EtBr staining. Images were captured by a Gel Doc XR+ Imaging Systems (Bio-Rad, USA).

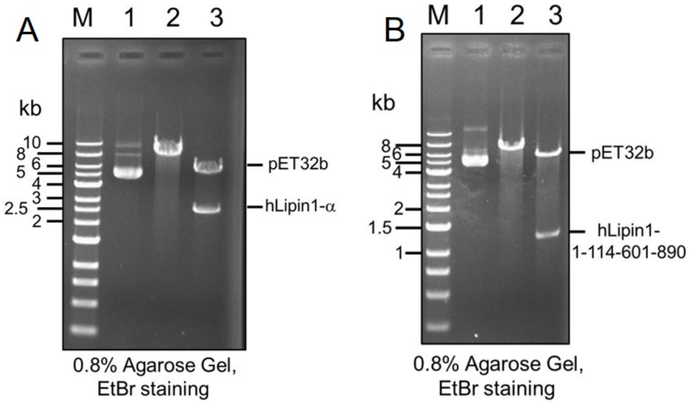

Figure S3. Confirm plasmid accuracy by restriction enzyme digestion. *A*, lane 1, pET32b-hLipin 1-α (1-890) plasmid only; lane 2, plasmid digested with *Eco*RI; lane 3, plasmid digested with *Kpn*I and *Xho*I. *B*, lane 1, pET32b-hLipin 1-α 1-114-601-890 plasmid only; lane 2, plasmid digested with *Eco*RI; lane 3, plasmid digested with *Kpn*I and *Xho*I. DNA fragments were separated by 0.8% agarose gel electrophoresis followed by EtBr staining. Images were captured by a Gel Doc XR+ Imaging Systems (Bio-Rad, USA).
